# A change in coral extension rates and stable isotopes after El Niño-induced coral bleaching and regional stress events

**DOI:** 10.1038/srep32879

**Published:** 2016-09-13

**Authors:** S. Hetzinger, M. Pfeiffer, W.-Chr. Dullo, J. Zinke, D. Garbe-Schönberg

**Affiliations:** 1 GEOMAR Helmholtz-Zentrum für Ozeanforschung Kiel, Wischhofstr. 1-3, 24148 Kiel, Germany; 2 Geological Institute, RWTH Aachen University, Wüllnerstrasse 2, D-52056 Aachen, Germany; 3 Curtin University of Technology, Dept. of Environment and Agriculture, Bentley, Western Australia 6102, Australia; 4 School of Geography, Archaeology and Environmental Studies, University of Witwatersrand, Johannesburg, South Africa; 5 Australian Institute of Marine Science, Nedlands, WA 6009, Australia; 6 Freie Universität Berlin, Institute for Paleontology, Malteserstrasse 74-100, 12249 Berlin, Germany; 7 Institut für Geowissenschaften, CAU Kiel, Ludewig-Meyn-Strasse 10, D-24118 Kiel, Germany

## Abstract

Coral reefs are biologically diverse ecosystems threatened with effective collapse under rapid climate change, in particular by recent increases in ocean temperatures. Coral bleaching has occurred during major El Niño warming events, at times leading to the die-off of entire coral reefs. Here we present records of stable isotopic composition, Sr/Ca ratios and extension rate (1940–2004) in coral aragonite from a northern Venezuelan site, where reefs were strongly impacted by bleaching following the 1997–98 El Niño. We assess the impact of past warming events on coral extension rates and geochemical proxies. A marked decrease in coral (*Pseudodiploria strigosa*) extension rates coincides with a baseline shift to more negative values in oxygen and carbon isotopic composition after 1997–98, while a neighboring coral (*Siderastrea siderea*) recovered to pre-bleaching extension rates simultaneously. However, other stressors, besides high temperature, might also have influenced coral physiology and geochemistry. Coastal Venezuelan reefs were exposed to a series of extreme environmental fluctuations since the mid-1990s, i.e. upwelling, extreme rainfall and sediment input from landslides. This work provides important new data on the potential impacts of multiple regional stress events on coral isotopic compositions and raises questions about the long-term influence on coral-based paleoclimate reconstructions.

Under projected anthropogenic climate change scenarios the widespread destruction of tropical coral reef ecosystems will become a reality over the next decades^1^. Current models forecast an increase in average global surface temperatures of at least 2 °C by 2100 relative to the pre-industrial period and up to 3–4 °C under existing patterns of human activity^2^. Around 30–50% of worldwide coral reefs have been largely or completely degraded due to the combined effects of local factors and global climate change during recent decades^3^. Water temperature is one of the most important variables limiting the growth and distribution of reef-building corals^4^. Shallow water tropical corals grow within a temperature range of around 18–30 °C^5^. Since the early 1980s sea surface temperatures (SSTs) have rapidly increased and tropical corals have experienced large-scale bleaching and mortality events^6^. During periods of sustained high temperature, bleaching occurs, a process during which the symbiosis between corals and their photosynthetic microalgae that live within their cells breaks down. Subsequently, the loss of the symbiotic algae causes whitening (“bleaching”) of the coral tissue^7^,^8^ and leads to their starvation, and in many cases to disease and mortality. For example, in 1998, following a super El Niño high temperature event, widespread coral bleaching and mortality was observed and approximately 16% of the worldwide coral communities bleached and died^8^. Climate-induced coral bleaching is one of the most serious threats to present-day coral reefs^9^.

The Caribbean was heavily impacted by El Niño-related bleaching events in 1995, 1997/98, 2005^10^ and 2010. Simultaneously, coastal Venezuelan reefs and marine communities were exposed to a series of extreme environmental fluctuations since the mid-1990s^11^, such as upwelling of cold and nutrient-rich waters leading to phytoplankton blooms, and increased terrestrial runoff, landslides and sedimentation due to recurring extreme rainfall events^12^,^13^,^14^,^15^. Over longer time scales (decades to centuries), however, it is difficult to assess how corals have responded to multiple climatic stressors due to the lack of historical data (e.g. underwater surveys). Long-term continuous records of past environmental variability and stress events would thus greatly help to better evaluate and quantify the potential implications and hazards of climate change on the development of coral reefs in the future.

Colonies of massive-growing tropical corals provide an ideal archive for the reconstruction of past environmental variability due to their continuous growth and long lifespan that can reach up to several centuries. The potential of massive-growing Atlantic corals (e.g. *Pseudodiploria, Orbicella, Siderastrea* spp.) to hold records of past climate change has been shown in several studies^16^,^17^,^18^,^19^,^20^,^21^,^22^,^23^,^24^,^25^. A recent study has demonstrated that bleaching events can be identified retrospectively in growth records from coral cores, allowing a comparison to existing local reef observations such as water quality and real-time bleaching reports^26^. However, we lack an understanding of how coral growth rates and skeletal geochemistry could be altered over time^27^ and what would be the implications for the accuracy of coral-based paleo-reconstructions.

Here, we present records of coral extension rates, stable isotopes and Sr/Ca ratios from a northern Venezuelan site (Fig. 1). Reefs at this coastal site were strongly impacted by coral bleaching following the super 1997–98 El Niño event. In addition, cold upwelling, high rainfall and runoff events pre-dated or directly followed the super 1997–98 El Niño and led to additional environmental stress^12^,^13^,^14^,^15^. Here, we aim to assess the impact of these multiple stressors on two corals. First, we compare geochemical proxy data from a *Pseudodiploria strigosa* coral to observation-based temperature to examine the ability of the coral to record climate variability. Second, we assess the influence of past El Niño-related warming events and local stress events on coral proxies in *P. strigosa*. Finally, we investigate the possible mechanisms causing a baseline shift in coral stable isotopes and extension rates in the uppermost years of the *P. strigosa* record and contrast growth records with those of a neighbouring *Siderastrea siderea* colony.

## Results and Discussion

### Coral proxy-temperature relationships

The coral δ^18^O and Sr/Ca proxy time series from core CHI7 (*P. strigosa*) extend from 1940–2004 and show similar interannual variability (Fig. 2a). Coral δ^18^O and Sr/Ca records display clear seasonal cycles (Fig. 2b) and the records are well correlated over the entire time period on monthly (r = 0.76, p < 0.001) and on mean annual scales (r = 0.69, p < 0.001). By comparing geochemical proxy data to observation-based temperatures, we examine the ability of the coral to record regional to large-scale climate variability: A direct comparison of coral δ^18^O to SST variability at the study site yields significant negative relationships (r = −0.52, p < 0.0001, 1941–2003, annual means, ERSST, Fig. 2a), confirming that coral δ^18^O records regional SST variability. Correlations are similar for coral Sr/Ca (r = −0.43, p < 0.001, Fig. 2a). Correlation coefficients are higher when comparing coral proxies to satellite SST data with higher spatial resolution (AVHRR, 1985–2004; Fig. 2b): for coral δ^18^O r = −0.57 (−0.46) and for coral Sr/Ca r = −0.68 (−0.60) for monthly (annual) means. In summary, both coral proxies record regional SST variability in the southeastern Caribbean (Fig. 2a).

### Influence of past El Niño-related warming events on SST and coral proxies

In Fig. 2a we show the influence of major El Niño events on the study site on interannual time scales by comparing coral proxy data to the Niño3.4 index, an index for the strength of SST anomalies in the tropical Pacific Niño3.4 region^28^. On an annual mean scale, both coral proxies display decreasing values during most major El Niño events indicating increased water temperatures at the study site (Fig. 2a). This is confirmed by instrumental data, which show regionally higher SSTs during most warming events related to El Niños (ERSST, Fig. 2a). These findings are in line with earlier results^17^,^29^ that described a similar impact of past El Niño events on coral proxies from a *P. strigosa* coral record in the southeastern Caribbean.

**Figure 1:**
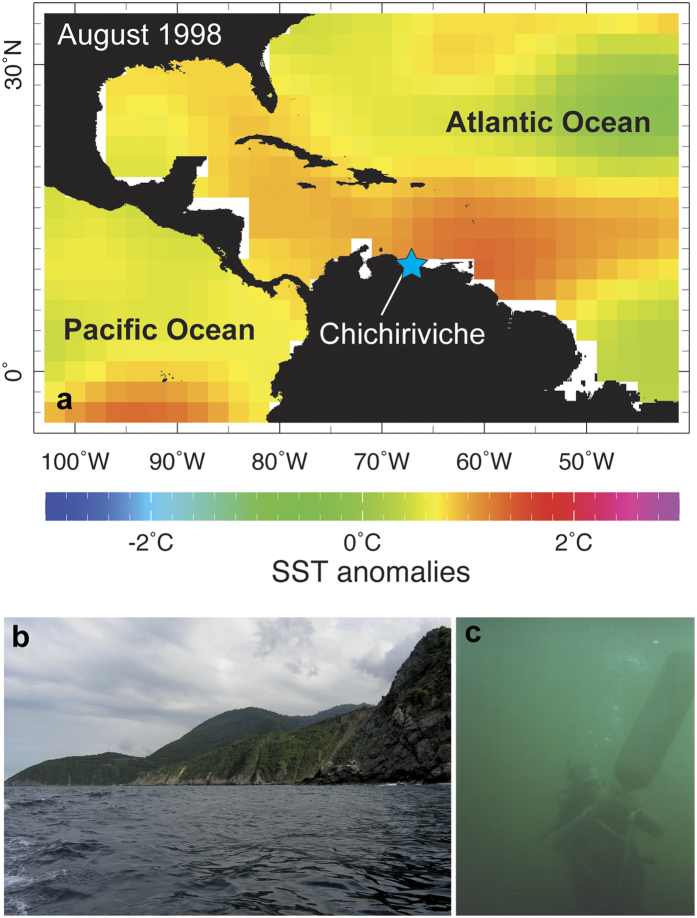
Overview map and conditions at the sampling site. (**a**) Caribbean SST anomalies during summer of 1998 (El Niño event). Blue asterisk marks the study site Chichiriviche de la Costa, Venezuela (10.55°N, 67.24°W). Data from Improved Extended Reconstruction of SST (ERSST.v3b) compilation. (**b**) View of the Chichirivichi coastline from the sampling site displaying high coastal relief. (**c**) Extremely poor underwater visibility was encountered during December 2004 coral sampling, probably due to high sediment input. The town was hit by a mud slide in the same week. Map (**a**) created using the free web application KNMI Climate Explorer (available at http://climexp.knmi.nl/).

**Figure 2:**
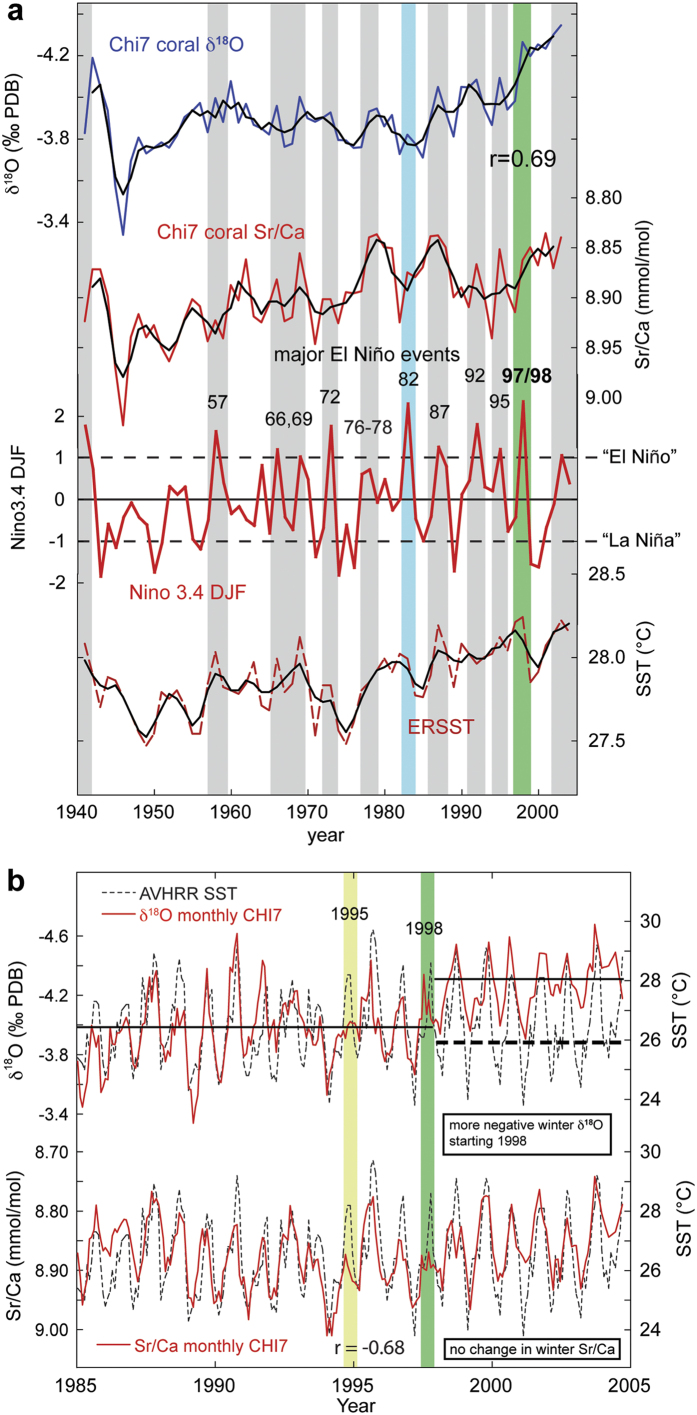
Geochemical coral proxies (δ^18^O, Sr/Ca) compared to major El Niño events and water temperature. (**a**) Niño3.4 DJF index (red), grey bars indicate major El Niño events; green bar highlights record 1997/98 event, blue bar - 1982 El Niño. SST (red dashed, 9–11°N, 67°W) from ERSST.v3b dataset^49^. Colored lines depict annual resolution, black lines 3-year running averages. (**b**) Monthly coral δ^18^O (top) and Sr/Ca (bottom) compared to high-resolution satellite SST for the study site. Monthly SST (dashed, 10.5–11°N, 67–67.5°W) derived from 4 km spatial resolution Advanced Very High Resolution Radiometer (AVHRR) Pathfinder Version 5 SST dataset (http://www.nodc.noaa.gov/sog/pathfinder4km/). Solid lines in upper panel depict the average coral δ^18^O before and after 1998, dashed bold line indicates limit of winter coral δ^18^O after 1998.

Most warm ENSO phases in the Niño3.4 index are identifiable in the coral δ^18^O (Sr/Ca) time series (e.g., the ENSO warm phases of 1966/69, 1972, 1987, 1992/95 and 1997/98). When comparing the response of coral proxies to *individual* events, focusing on the El Niños of 1982/83 and 1997/98 (Fig. 1), the two strongest events of the past 50 years, we note that in 1982/83 coral proxies and SST data point to only a weak impact at the study site. Instrumental SST data only show moderate warming, coral proxies suggest no significant warming (Fig. 2a). However, although the 1982/83 El Niño event in the Pacific was of similar strength as the 1997/98 event, it was of shorter duration and only very little coral bleaching was reported in the Caribbean as water temperatures stayed below the record highs of 1997/98^30^. Additionally, there is a cooling impact in 1982 due to strong remote forcing from high levels of atmospheric aerosols after the El Chichón volcanic eruption (Mexico) in the same year that has been linked to reduced SSTs^31^. The 1997–98 El Niño was the strongest event on record to date, resulting in wide-spread coral bleaching worldwide, also in the southern Caribbean, the studied region^30^. Both coral proxies and regional SST data indicate significant warming in the same years in mean annual data (Fig. 2a).

### Baseline shift in stable isotopes and coral extension rates

#### Oxygen isotopes

Coral proxy data were analyzed in monthly resolution. In Fig. 2b, we take a closer look at the relationship between both coral proxies and high-resolution satellite SST data (AVHRR SST), which are available for the last 20 years of the coral record. Both coral δ^18^O and Sr/Ca follow seasonal temperature variability at the study site closely (Fig. 2b). However, from 1998 onwards, coral δ^18^O seasonal range decreases by approximately 40% (seasonal range δ^18^O: 1985–1997, 1.28‰; 1998–2004, 0.77‰) due to a baseline shift to more negative winter δ^18^O values (Fig. 2b). At the same time, mean coral δ^18^O changes from −3.98‰ (1985–1997) to −4.28‰ (1998–2004), while no significant change is observed in mean Sr/Ca. This shift occurs after the 1997/98 record El Niño event that caused bleaching of corals worldwide, including Caribbean reefs^10^. Due to the shift in winter δ^18^O, which persists until the end of the coral record, annual means are more negative (by 0.3‰) for the time period after the event (Fig. 2b, 1999–2004). This shift in the mean would translate to a change of ~1.5 °C in SST when converting coral δ^18^O to temperature using previous calibration equations for *P. strigosa* (0.2‰/1 °C^29^). However, no shift is observed in winter coral Sr/Ca values, Sr/Ca seasonal range, or in seasonal SST over the same time period (Fig. 2b). Thus, changing temperature is ruled out as the cause for this abrupt shift in winter coral δ^18^O. When examining correlations of coral δ^18^O to instrumental SST (AVHRR) for the time periods before and after the 1998 shift, we observe that the correlation coefficients decrease markedly after 1998 (e.g. r = −0.62, 1985–1998; r = −0.28, 1998–2003, annual means). This suggests that coral δ^18^O stops to accurately representing seasonal water temperature variability due to the baseline shift in δ^18^O winter values after 1998. A potential cause for such a shift might be a change in microsampling technique, sampling resolution or reduced winter growth. However, we exclude this possibility as both proxies were analyzed on the same sample material and there is no similar effect displayed in coral Sr/Ca (Fig. 2b).

The shift in coral δ^18^O coincides with the mass bleaching event of 1997/98, which was caused by a record El Niño event in the tropical Pacific that produced anomalously warm ocean temperatures. In the recent past, increasingly severe coral bleaching events occurred in the Caribbean causing coral decline and mortality, e.g. in 1995, 1998, 2005, and 2010^10^,^32^. In the southern Caribbean, prolonged and extreme heating events negatively affected coral reef communities at several sites along the northern Venezuelan coast, mainly documented in the Morrocoy National Park (MNP), where the CARICOMP (Caribbean Coastal Marine Productivity Program) surveys started in 1993^33^. Although no data are available directly for our study site, data from nearby sites indicate a strong impact and declining reef health starting in the mid-1990s, with a decreasing trend in coral cover reported for MNP (west of Chichiriviche; from 55% in 1996 to 39% in 2011) and Los Roques National Park (north of Chichiriviche; from 44% in 1999 to 25% in 2012)^30^. High and persistent SSTs lead to thermal stress on corals, which as a result might also impair the coral’s ability to record the full scale of seasonal warming^34^. We observe a dampening of the seasonal amplitude in both geochemical proxies in 1995 and 1998, although in 1998 the reduction is more pronounced in coral Sr/Ca. This is documented by reduced summer values in both years (Fig. 2b). The dampening in 1995 directly corresponds to observations in the MNP, where widespread coral bleaching was reported in late 1995, with more than 75% of coral colonies (up to 90% in the dominant massive-growing species *Orbicella faveolata*) bleached, partially bleached, or pale^14^.

#### Coral extension rates, carbon isotopes and regional stress events

**Figure 3:**
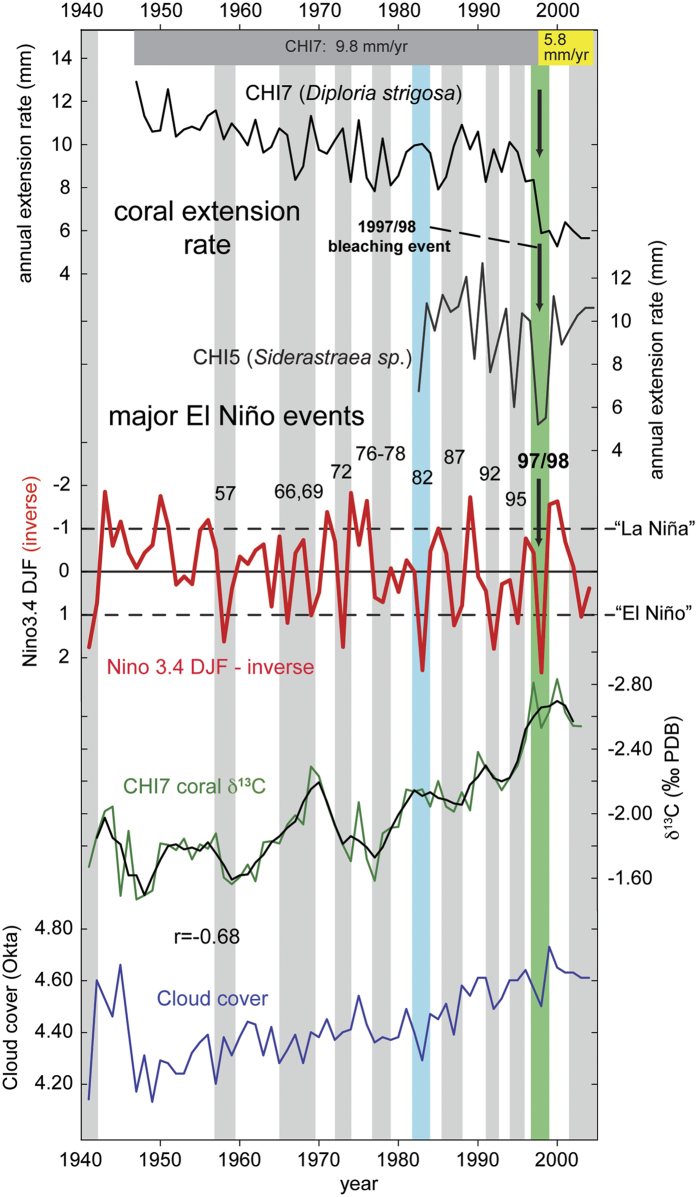
Coral annual extension rates and their relation to major El Niño events. Note: Niño3.4 DJF index (red) plotted inversely, grey bars indicate major El Niño events; green bar highlights record 1997/98 event. Extension rates derived from analysis of X-radiographies of coral slabs (cores CHI 5 and 7). All data in annual resolution. Grey and yellow bars in top panel show average annual extension rates for core CHI7 for the respective time intervals. Lower plots: Coral δ^13^C; Cloud cover (10–14°N, 66–68°W, ICOADS data by NOAA/OAR/ESRL PSD, http://www.esrl.noaa.gov/psd/).

Ocean warming and thermal stress events can also influence coral growth and previous studies have reported decreasing linear extension rates in corals due to rising SSTs^35^ and during El Niño events^7^,^36^. Here, annual skeletal extension rates were measured based on high-resolution x-ray images displaying coral density banding. In addition to core CHI7 (*P. strigosa*), we have analyzed linear extension rates on a second coral core from the same site, but from a different coral species (core CHI5, *S. siderea*). Although this core is shorter in length than core CHI7, it yields important information in its record (Fig. 3). Coral annual extension rates indicate a growth slowdown during major El Niño events. For instance, distinct reductions in extension rate are observed in core CHI5 in El Niño years, e.g. 1982, 1992, 1995 and 1998, with the strongest reduction during the largest El Niño event (1998). Core CHI7 only shows a significant reduction in 1998, but the extension rate remains on this much lower level afterwards (Fig. 3). The strongest reduction in annual extension rates occurs in 1998 in both cores. However, while reduced extension rates persist (5.8 mm/yr) from 1998 onwards in CHI7 (*P. strigosa*), extension rates return to pre-1998 values (~10 mm) in core CHI5 (*S. siderea*) (Fig. 3). The fact that extension rates after 1998 stay on a level that is much lower than in the entire earlier record, concurrent with the distinct baseline shift in coral δ^18^O, implies that a major change took place in the CHI7 coral colony in the mid-1990s.

**Figure 4:**
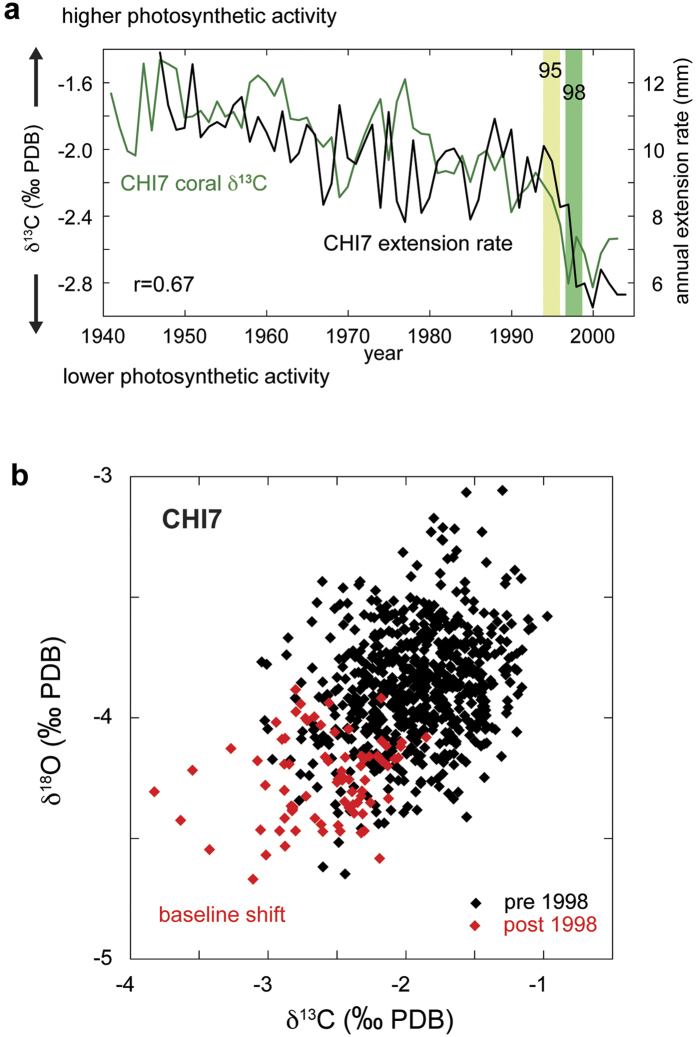
Coral δ^13^C, δ^18^O and annual extension rates. (**a**) Annual coral δ^13^C (green) from coral core CHI7 and extension rate (black). Light (dark) green bar highlights 1995 (1998) El Niño event. Note: δ^13^C plotted inversely compared to Fig. 3. (**b**) Coral δ^13^C versus δ^18^O for core CHI7. Data from before the 1998 bleaching are black, and data from 1998 onwards are red.

In addition to the observed baseline shifts in coral δ^18^O and extension rates in core CHI7, a further line of evidence for a possible change in coral physiology in this colony comes from coral δ^13^C that was measured over the entire record along with δ^18^O and Sr/Ca ratios (Figs 3 and 4). Coral δ^13^C in symbiotic reef corals is believed to be predominantly influenced by metabolic fractionation effects^37^,^38^,^39^. However, the long-term dynamics in coral physiology after recovery from bleaching are complex^40^. Environmental variables influencing coral metabolism such as light, the main driver of photosynthesis, and abundance of zooplankton have been shown to play an important role in coral skeletal δ^13^C variations^41^.

When directly comparing coral δ^13^C and extension rates in core CHI7, we find a significant positive relationship and a similar overall trend (r = 0.67, p < 0.001; Fig. 4a). The long-term depletion of the δ^13^C record is consistent with the anthropogenic δ^13^C-Suess effect, i.e. the burning of isotopically “light” fossil fuels, which decreases the δ^13^C value of carbon reservoirs. However, a distinct swing in δ^13^C to more negative values in the mid-1990s, lower than in the entire previous coral record, may hint at a change in coral physiology. Starting after the 1995 El Niño event and continuing to around the year 2000, this remarkable shift to lower coral δ^13^C coincides with drastically reduced extension rates (CHI7) and the baseline shift in coral δ^18^O (Figs 2b, 3 and 4a). A direct comparison using coral δ^13^C and δ^18^O values before and after 1998 visualizes this isotopic baseline shift (CHI7, Fig. 4b). At the same time, local SSTs did not change significantly (Fig. 2) and extension rates in a neighboring coral colony (CHI5, *S. siderea*; Fig. 3) return to pre-1998 rates. However, we note that the extension rate observations are based on only one colony from each species at this site. Clearly, a larger sample number is necessary to investigate species-specific effects. Figure 4b shows that both δ^18^O and δ^13^C in CHI7 (*P. strigosa*) are more depleted after the shift in 1998. This is remarkably similar to the results of Carilli *et al*.^19^ (their Fig. 6). We cannot explain these observed isotopic baseline shifts with kinetic isotopic effects, as in this case, slower coral growth after 1998 should lead to more positive δ^18^O and δ^13^C values. Also, low growth rates should additionally influence coral Sr/Ca ratios and shift them towards larger ratios^18^, but our Sr/Ca ratios are not affected. We also cannot explain our results with sampling problems, as sampling off the central axis of the thecal walls in *P. strigosa* should also result in more positive δ^18^O and δ^13^C values, and larger Sr/Ca ratios (see Giry *et al*.^18^, their Fig. 5).

We also researched other possible explanations for this baseline shift. For example, cloud cover data do not show significant changes after 1998 (Fig. 3). Thus, a change in light levels/solar irradiance, that could potentially influence photosynthesis, is ruled out. Observations from the MNP, west of the study site, suggest additional influence from other stressors on coastal Venezuelan reefs starting in the mid-1990s: A mortality event in early 1996 in the MNP was described as one of the greatest massive die-offs reported in Venezuela. It has been attributed to an abnormal upwelling and expansion of cold (<20 °C) and nutrient-rich water along the central-west coast, combined with a lack of winds that produced a plankton bloom leading to anoxic conditions on the seafloor^14^. Detailed surveys in the MNP revealed that corals suffered mortalities from 60 to 98%, depending on the location, just one year after the 1995 El Niño-related bleaching event. However, due to the distance between the sites, not all stress events can be directly compared between sites. Some massive-growing corals at MNP (*Orbicella annularis* spp. complex) showed different rates of bleaching, while corals of the species *S. siderea* were reported to not suffer any obvious damage^14^. This is noteworthy as, unlike core CHI7 (*P. strigosa*, also a massive-growing coral species), extension rates in core CHI5 (*S. siderea*) swiftly recover to previous values after decreases in 1995 and during 1997/98 (Fig. 3), corresponding to the lesser impact of stress on this species reported at nearby MNP^14^. These results are similar to those of a recent study, which found no impact on extension rates of backreef and nearshore colonies of *S. siderea* (Belize) by thermal stress events^42^.

Another major stress event took place in December 1999, when Venezuela saw its highest monthly rainfall in 100 years, triggering massive landslides, debris flows and flooding along the northern coast of Venezuela. This extreme event, which killed more than 30,000 people and caused massive damage to communities and infrastructure^15^, has also severely influenced local coral reefs, including the study site (confirmed by local observations). At MNP, this record rainfall event led to a distinct decrease of salinity, which lasted for over a month, impacting shallow water environments even one year after the impact^12^ and increasing terrestrial runoff and sedimentation which reduced visibility. Coral reefs in the affected area have seen a steep decline after this event^13^.

In summary, coastal Venezuelan reefs and marine communities were exposed to a series of extreme environmental fluctuations since the mid-1990s, such as high-temperature episodes related to El Niño events, upwelling of cold and nutrient-rich waters leading to phytoplankton blooms, and increased terrestrial runoff, landslides and sedimentation due to recurring extreme rainfall events. These events lead to episodes of coral die-offs and widespread mortalities in marine communities^14^, adding up to an interplay of mounting pressures and decline of coastal Venezuelan marine ecosystems.

Our results from both cores provide evidence for coral responses to increased influence of different stressors and extreme events. The fact that both stable isotopes and extension rates in core CHI7 show a distinct baseline shift in the mid-1990s, which is not evident in growth data of core CHI5 from a different species, points to a major change in the physiology of coral CHI7 (*P. strigosa*). Recent studies have shown that in reef-building corals, some genotypes of endosymbiotic algae are more susceptible to heat stress than others, corals harboring these types are less resistant to temperature-induced bleaching^43^. A change to more heat-tolerant symbiont types after severe bleaching and mortality events has also been reported^44^. As symbiont types differ in relative photosynthetic rates and efficiency and significantly influence coral growth^45^, physiological differences may also have varying effects on geochemical signature, isotopic chemistry and coral calcification mechanisms.

However, a change in coral symbiont genotype over time is very difficult to detect in coral records and few studies have investigated whether such shifts can have an effect on coral geochemistry. A recent study has suggested that a change in coral symbiont clade community might be the driver of an observed shift in oxygen and carbon isotopic composition and extension rates in coral cores from nearby Belize^19^. The main findings of this study are remarkably similar to the changes and timing observed in core CHI7. They have found: (i) baseline shifts in the stable isotopic composition of two corals (*O. faveolata*) after the 1995 and 1998 bleaching events of similar magnitude as observed here (0.3‰ mean difference between pre- and post-bleaching in δ^18^O), (ii) unaffected coral Sr/Ca ratios, and (iii) growth anomalies during both bleaching events. However, a change to a more heat-resistant symbiont clade after bleaching might be only one possible explanation for the observed changes in core CHI7, but we have no direct evidence as live coral tissue was not sampled for DNA analysis.

Our findings have major implications for the interpretation of coral-based records and paleotemperature reconstructions, especially if these studies rely on coral stable isotopes. Our results suggest that after a major change in coral physiology seasonal stable oxygen variability is dampened and doesn’t accurately represent seasonal water temperature changes. Assuming that such baseline shifts in stable isotopes have occurred in the past after major stress events and, so far, have remained undetected in longer, often century-long coral proxy time series, a dampened seasonal cycle in coral δ^18^O might lead to inaccurate paleo-reconstructions over certain time periods within a record. We have observed a reduction in annual coral extension rate coinciding with shifts in stable isotopes (δ^18^O and δ^13^C). Thus, in other coral records the existence of a significant prolonged period of change in extension rate (either increase or decrease) and coral δ^13^C might be an indirect marker for a potential physiological change. Especially sections of coral records displaying distinct stress bands, that can be related to past bleaching events, should be scrutinized for potential long-term changes in coral extension rates and consequently for changes in coral isotopic signatures in future studies. However, further testing using more coral records from a larger number of sites in combination with sampling of live tissue is critical to better understand how physiological changes in massive corals may influence coral-based proxies for environmental change.

## Methods

### Coral sampling

Cores CHI7 (*P. strigosa*) and CHI5 (*S. siderea*) were recovered in December 2004 from hemispherically-growing coral colonies in a fringing reef located near Chichiriviche de la Costa, Venezuela (10.55°N, 67.24°W; Fig. 1). Both cores were drilled vertically in 5 m water depth using SCUBA. Cores have a length of 0.56 m (CHI7) and 0.24 m (CHI5) with a diameter of 36 mm. Both cores were sectioned longitudinally into 7 mm thick slabs, then x-rayed to expose annual density band couplets. Chronologies were generated by counting the well-developed annual density bands. Core CHI7 extends continuously from 1940 to 2004, core CHI5 extends from 1982 to 2004. Skeletal extension rates were measured from annual density bands displayed in the x-radiographies using greyscale image analysis software (ImageJ) and average 9.3 mm/year (±1.71 mm, core CHI7) and 9.5 mm/year (±2.04 mm, core CHI5). Extension rates serve as a proxy for calcification rates along the central axis of the dense thecal walls of *Pseudodiploria* corals.

### Coral proxies and chronology

Core CHI7 was sampled for stable isotope and trace element analysis. Powdered samples were collected using a low-speed micro drill with a 0.7 mm diameter drill bit. The slabs were sampled continuously along the corallite walls (theca) in order to avoid mixing of sample powder from different skeletal elements. Samples were retrieved at approximately 0.8 mm intervals, yielding on average 10–12 samples per year. The powdered samples were split into separate aliquots for stable isotope (δ^18^O, δ^13^C) and trace element (Sr/Ca) analysis. Sr/Ca was measured with an ICP-OES at the University of Kiel following the techniques described by Schrag^46^ and de Villiers *et al*.^47^. Average analytical precision of Sr/Ca determinations is 0.09% RSD or <0.01 mmol/mol (1σ) (n = 729; 1 standard after every 6 samples) from multiple measurements on the same day and on consecutive days. δ^18^O and δ^13^C were analyzed using a Thermo Finnigan Gasbench II Deltaplus at GEOMAR. The isotopic ratios are reported in ‰ VPDB relative to NBS 19, and the analytical uncertainty is less than 0.06‰ (1σ) (n = 729; 2 standards after every 10 samples).

The age model was established based on the pronounced seasonal cycle in the Sr/Ca record. The maximum (minimum) Sr/Ca was tied to March (September), which is on average the coolest (warmest) month in the study area. Coral δ^18^O, δ^13^C and Sr/Ca timeseries were linearly interpolated between these anchor points using the Analyseries software^48^ to obtain monthly proxy timeseries. The uncertainty of the age model is approximately 1–2 months in any given year.

## Instrument-derived data

Local *in situ* records of water temperature are not available for the study site. Thus, monthly SSTs for the 2° latitude by 2° longitude box including the coral site (9–11°N, 67°W) were extracted from the Improved Extended Reconstruction of SST (ERSST.v3b) compilation for the same time period^49^. The ERSST uses statistical methods to fill in gaps in the instrumental data base^49^. High-resolution satellite SST data for the study site (10.5–11°N, 67–67.5°W) were retrieved from the 4 km spatial resolution Advanced Very High Resolution Radiometer (AVHRR) Pathfinder Version 5 dataset (http://www.nodc.noaa.gov/sog/pathfinder4km/). These data were provided by GHRSST and the US National Oceanographic Data Center^50^. The AVHRR and ERSSTv.3b datasets are highly correlated on monthly (r = 0.96, p < 0.0001) and annual mean scales (r = 0.94, p < 0.0001) for the overlapping time period (1985–2004).

## Additional Information

**How to cite this article**: Hetzinger, S. *et al*. A change in coral extension rates and stable isotopes after El Niño-induced coral bleaching and regional stress events. *Sci. Rep.* **6**, 32879; doi: 10.1038/srep32879 (2016).
